# Real-world clinical outcomes under intravenous and subcutaneous esketamine protocols for treatment-resistant depression: a multicenter, retrospective study

**DOI:** 10.3389/fpsyt.2026.1963841

**Published:** 2026-10-16

**Authors:** Gilberto Sousa Alves, Humberto M. M Dos Santos, Samira D. Damasceno, Laura M. Maciel, Otavio André Farias Sousa da Silva, Ciro Sousa de Moura Fe, Jilmario G. Santana, Mariélia Barbosa Leal de Freitas, André Férrer Carvalho, Dennison Monteiro

**Affiliations:** 1 Translational Psychiatry Research Group, Federal University of Maranhão, São Luís, Brazil; 2 Translational Psychiatry Research Group, Universidade do Porto, Porto, Portugal; 3 Translational Psychiatry Research Group, Centro Universitario Unidade de Ensino Superior Dom Bosco, São Luís, Brazil; 4 Translational Psychiatry Research Group, Deakin University Geelong Clinical School, Geelong, Australia; 5 Translational Psychiatry Research Group, Universidade de Pernambuco, Recife, Brazil

**Keywords:** esketamine, interventional psychiatry, intravenous administration, retrospective chart review, subcutaneous administration, treatment-resistant depression

## Abstract

**Background:**

Esketamine is a promising therapeutic option for treatment-resistant depression (TRD). Intravenous (IV) administration is the most extensively studied parenteral route. However, subcutaneous (SC) administration may offer simpler, more scalable delivery. Multicenter real-world evidence comparing IV and SC routes of administration remains limited.

**Objective:**

To report clinical outcomes and tolerability of parenteral esketamine protocols for TRD at three Brazilian advanced-care centers.

**Methods:**

This multicenter retrospective chart review study (2023–2025) included 72 outpatients aged 18–65 years who received esketamine 2 to 3 times weekly via IV (n = 26; 6 sessions) or SC (n = 46; 12 sessions) administration. Data were analyzed in RStudio 2026.01.0 Build 392 using R 4.5.2. The primary outcome was categorical response on the Beck Depression Inventory-II (BDI-II) at the shared 6-session milestone and at each protocol-specific endpoint. Secondary outcomes included continuous changes in BDI-II and Beck Anxiety Inventory (BAI) scores in the full sample, tolerability and documented adverse events, and exploratory factor analysis of baseline symptom dimensions.

**Results:**

After six sessions, BDI-II response distribution favored the IV protocol, although the between-group difference was not statistically significant (Pearson’s chi-square = 5.661; p = 0.059; odds ratio, 3.06; 95% CI, 1.22–8.00). At the protocol-specific endpoints, categorical BDI-II response did not differ significantly between groups (Pearson’s chi-square = 1.227; p = 0.541; odds ratio, 1.44; 95% CI, 0.57–3.73). No serious adverse events or adverse-event-related discontinuations were reported; dissociation was more frequent with IV than with SC treatment (53.8% vs. 13.0%; p = 0.001), whereas nausea and mild transient blood pressure increases did not differ significantly. BDI-II factor analysis identified cognitive-affective and somatic-neurovegetative dimensions.

**Conclusions:**

The IV protocol showed a more favorable BDI-II response distribution at the shared 6-session milestone, whereas SC esketamine achieved statistically comparable categorical outcomes at its 12-session protocol-specific endpoint. Route selection should consider speed of response, tolerability, dosing, operational complexity, and access. SC esketamine may be a feasible option where simplicity, tolerability, and access are priorities. Results should be interpreted with caution because of the retrospective, observational study design.

## Introduction

Treatment-resistant depression (TRD) remains a major challenge in psychiatry. It is generally defined as inadequate improvement after at least two adequate antidepressant trials, although definitions vary (1–3). TRD is associated with functional impairment, chronicity, and suicide risk (1, 4–6). Remission rates remain limited even with evidence-based pharmacotherapy, highlighting the need for faster and more effective therapeutic options (1, 7).

Ketamine and esketamine have advanced depression treatment through rapid, non-monoaminergic antidepressant effects (1, 7, 8). Their effects may involve N-methyl-D-aspartate (NMDA) receptor antagonism (9), glutamatergic signaling, synaptic plasticity, and neuroactive metabolites (10, 11). Unlike conventional antidepressants, ketamine-based treatments can reduce symptoms within hours to days, which is especially relevant in severe or suicidal presentations (4, 5, 12). Some studies have also implied a role for opioid neurotransmission, as ketamine also appears to activate the mu, kappa, and delta-opioid receptors (13–15). However, questions remain about route, durability, dosing, comparative effectiveness, and implementation (1, 7). Intranasal esketamine has the strongest formal regulatory support. In Brazil, Brazilian Health Regulatory Agency (ANVISA) approved it in 2020 for adults with major depressive disorder who failed at least two antidepressants, and for rapid symptom reduction in major depression with acute suicidal ideation or behavior, both in combination with oral antidepressants (16, 17). Even so, cost, limited availability, and supervised-administration requirements restrict access (7, 17, 18).

Recent best-practice recommendations also emphasize protocolized, supervised, measurement-based care and individualized assessment of response and tolerability for ketamine-based interventions (19). Most evidence for ketamine/esketamine in depression comes from intravenous (IV) administration, the best-established parenteral route (1, 7, 20–23), whereas fewer studies have investigated subcutaneous (SC) esketamine (24–27). Studies comparing IV and SC esketamine protocols, including real-world multicenter data, remain limited and heterogeneous (7, 23–28). Repeated IV protocols have shown meaningful response and remission in controlled and real-world studies (23, 29). However, IV delivery requires pumps, trained staff, venous access, and monitoring, which may limit scalability in routine and lower-resource settings (7, 24). The SC route has gained interest as a simpler alternative (24, 25). In an open-label dose-titration study, eight weekly sessions of SC esketamine were associated with clinically meaningful reductions in depressive symptoms, with reported response and remission rates of 52.17% and 34.78%, respectively (30). Current studies suggest that SC ketamine/esketamine is feasible, well tolerated, and clinically effective, with lower infrastructure demands than IV administration (24, 25). In practice, route selection also depends on tolerability, convenience, venous access, staffing, and service organization (18, 24).

The Brazilian Psychiatric Association has specifically addressed bioethical responsibilities in its guidance on off-label procedures in psychiatry (31). Off-label use refers to medication use outside the approved indication, route, dosage, or population (31–33). Although intranasal esketamine is approved in Brazil, IV and SC routes of administration remain off-label and therefore require physician responsibility, appropriate monitoring, scientific justification, and ethical caution (16, 31, 32, 34). These uncertainties are particularly relevant in routine psychiatric practice, where clinical decisions must balance efficacy, access, infrastructure, and tolerability (18, 23, 29). Off-label ketamine/esketamine may be justified in refractory cases, but it requires transparent informed consent, careful risk-benefit assessment, and institutional safeguards (31, 32).

Thus, this study aimed to report the clinical outcomes and tolerability of IV and SC esketamine protocols across three specialized Brazilian centers: a 6-session IV protocol delivered at two participating centers and a 12-session SC protocol delivered at one participating center. We also assessed response at the common 6-session time point and explored baseline symptom dimensions. We hypothesized that both modalities would be effective but would differ in response timing and implementation-relevant characteristics.

## Methods

### Study design and participating centers

This multicenter retrospective, observational chart review used data from patients treated with parenteral esketamine at three private Brazilian advanced-care centers between 2023 and 2025: 1) *Monteiro Institute of Neuromodulation and Interventional Psychiatry* (IMONN), Recife; 2) *Tau Medicina Esketamine Infusion Center*, São Luís; and 3) *Rondônia Center for Interventional Psychiatry and Neuromodulation*, Ji-Paraná. One participating center administered SC treatment (Tau Medicina), while two centers administered IV treatment. Because the administration route was center-dependent and the treatment schedules differed, the study compared center-specific routine-care protocols rather than standardized experimental interventions or an isolated causal effect of administration route. Selected routine-care practices across centers—including psychometric assessment tools, primary outcomes, dose calculation and titration strategies, and vital-sign monitoring—were harmonized before baseline through collaborative scientific meetings involving experts (G.S.A., H.M., D.M.) in 2021 and 2022.

### Sample and eligibility criteria

The final analytical sample included 72 adults aged 18–65 years: 26 treated under the IV protocol and 46 under the SC protocol. Eligible patients had a depressive disorder established in routine psychiatric practice according to DSM-5, an indication for parenteral esketamine after insufficient response to previous treatment, and sufficient baseline and endpoint data for the planned analyses. For retrospective eligibility assessment, we operationally defined treatment resistance as inadequate improvement after at least two antidepressant treatment trials considered adequate during the current depressive episode, taking documented dose, treatment duration, and adherence into account (1, 2). For treatment-resistant bipolar depression, we adopted the definition proposed by Fornaro and colleagues (2): lack of response to at least two established treatments for bipolar depression, including agents such as the olanzapine–fluoxetine combination, brexpiprazole, cariprazine, quetiapine, or aripiprazole. We determined eligibility from the treatment history documented in the clinical records. Because treatment histories were derived from routine electronic medical records, detailed information on the exact antidepressant agent, dose, duration, adherence, augmentation strategy, and reason for failure followed medical decisions across all participating centers. **Supplementary Data** depict oral treatment information for each center.

Records were excluded for insufficient data, incompatible protocols, duplication or inconsistency, unconfirmed esketamine indication, relevant cardiovascular or neurological disorders, functionally significant autoimmune rheumatologic disease, severe obesity, or substance dependence.

The retrospective screening process initially identified 450 clinical records across the participating centers. After initial screening and review of the available clinical documentation, 126 records remained for further eligibility assessment (**Figure 1**). Thus, 324 records were not retained at this stage. Because exclusion reasons were not prospectively recorded in a standardized screening log, individual and mutually exclusive reasons for these 324 records could not be reliably reconstructed from the retrospective source data.

**Figure 1:**
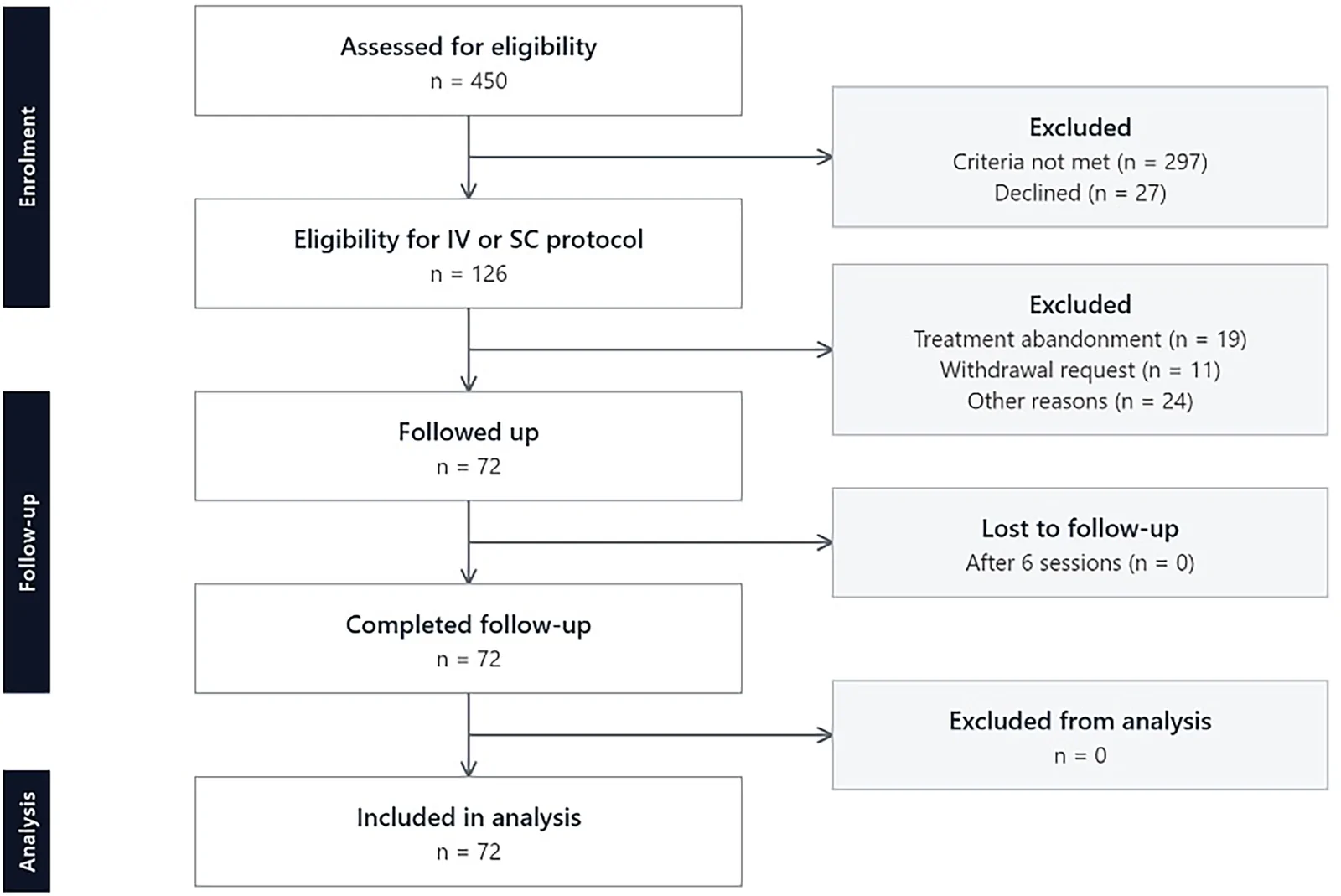
Flowchart showing the number of medical records initially identified, the cases excluded after screening and final sample included for analysis. Treatment abandonment refers to abandonment of the ketamine protocol, with subsequent maintenance of conventional oral treatment.

Among the 126 records retained after initial screening, 54 did not contribute to the final analytical cohort. Available records classified these cases as treatment abandonment (n = 19), withdrawal request (n = 11), or interruption for other reasons (n = 24), including treatment-session nonattendances or lack of the required companion (**Figure 1**). These cases were therefore not included in the efficacy analyses requiring the predefined baseline and endpoint assessments.

### Sociodemographic and clinical characterization

Extracted variables, including socio-demographic, clinical characterization and treatment response, are depicted in **Table 1**. We retained primary diagnoses according to the categories documented in the source clinical records and reported them separately by treatment protocol (**Table 1**). Due to the naturalistic design, previous and concomitant pharmacological regimens varied across patients and included antidepressants and/or mood stabilizers. We based treatment-resistance status on the eligibility definition described above and on the treatment history documented in routine clinical records, rather than on a prospectively standardized treatment-resistance staging instrument.

**Table 1:** Baseline sociodemographic and clinical characteristics of the sample.

Variable	Total (n = 72)	IV (n = 26)	SC (n = 46)	Test statistic	df	p-value
Age, years	36.9 ± 13.7	36.9 ± 13.7	36.9 ± 14.4	U = 576	–	0.801
Sex						
Male	29 (40.3%)	11 (42.3%)	18 (39.1%)			
Female	43 (59.7%)	15 (57.7%)	28 (60.9%)	χ² = 0.000	1	0.989
Mood disorders (MD)						
Bipolar depressive episode	18 (25%)	4 (15.4%)	14 (30.4%)			
Unipolar depressive episode^2^	54 (75%)	22 (84.6%)	32 (69.6%)	χ² = 2.010	1	0.157
MD with no psychiatric comorbidities	33 (45.8%)	10 (38.5%)	23 (50.0%)	–	–	–
BDI-II Score						
Baseline	38.5 (30.5-46.5)	38.5 (30.0-47.0)	38.5 (31.0-46.0)	–	1	0.640
BAI Score						
Baseline	25.0 (12.0-37.0)	28.50 (14.0-37.0)	25.0 (11.0-36.0)	–	1	0.521
Personality disorders						
Borderline personality disorder	10 (13.9%)	2 (7.7%)	8 (17.4%)	χ² = 1.307	1	0.314^1^
Obsessive-compulsive personality disorder	2 (2.8%)	1 (3.8%)	1 (2.2%)	χ² = 0.172	1	0.411^1^
Other personality disorders	7 (9.7%)	4 (15.4%)	3 (6.5%)	χ² = 1.487	1	1.000^1^
Other comorbidities						
Generalized anxiety disorder	24 (33.3%)	14 (53.8%)	10 (21.7%)	χ² = 6.329	1	0.012
Fibromyalgia	1 (1.4%)	0 (0.0%)	1 (2.2%)	χ² = 0.573	1	1.000^1^
Social phobia	2 (2.8%)	0 (0.0%)	2 (4.3%)	χ² = 1.163	1	0.523^1^
Premenstrual dysphoric disorder	1 (1.4%)	1 (3.8%)	0 (0.0%)	χ² = 1.794	1	0.357^1^
Clinical history						
Previous suicide attempt	45 (62.5%)	16 (61.5%)	29 (63.0%)	χ² = 0.000	1	1.000
Previous psychiatric hospitalization	11 (15.3%)	4 (15.4%)	7 (15.2%)	χ² = 0.000	1	1.000^1^
Adverse events						
Dissociation	20 (27.8%)	14 (53.8%)	6 (13.0%)	χ² = 11.826	1	0.001
Nausea	9 (12.5%)	2 (7.7%)	7 (15.2%)	χ² = 0.860	1	0.486^1^
Transient blood pressure elevation	4 (5.6%)	1 (3.8%)	3 (6.5%)	χ² = 0.227	1	1.000^1^

Continuous variables are presented as mean ± standard deviation; categorical variables are presented as n (%). P values were obtained using the Mann–Whitney U test or Pearson’s chi-square test for continuous or categorical data, respectively. ^1^P value estimated using Monte Carlo simulation because expected cell counts were <5. IV, intravenous; SC, subcutaneous;^2^only recurrent episodes of unipolar depression computed; NA, not applicable. Event labels reflect terminology supported by the available routine-care documentation. Severity grading was not inferred when not explicitly recorded.

### Therapeutic protocols

All centers used injectable esketamine hydrochloride (50 mg/mL). The IV protocol consisted of six esketamine sessions administered 2 to 3 times weekly according to the routine clinical schedules of the participating IV centers. Esketamine was diluted in 250 mL of 0.9% sodium chloride solution and infused over 1 hour via peripheral venous access in an upper limb. The SC protocol consisted of 12 esketamine sessions administered 2 to 3 times weekly, with esketamine administered subcutaneously in the hypogastric region.

Dose titration for both IV and SC protocols started at 0.5 mg/kg, with increases to 0.75 mg/kg and then 1.0 mg/kg in sessions where adequate response—defined as ≥50% symptom improvement or remission—had not been achieved. During each dosing session, all patients were individually accompanied by a nurse and remained in a reclining chair. Hemodynamic monitoring, including blood pressure, heart rate, and oxygen saturation, was performed every 10 minutes. Patients remained under clinical observation for 1 hour or longer after administration, when appropriate. Management of clinically relevant blood-pressure elevations, dissociation, nausea, or other acute effects followed the routine-care procedures of each participating center.

### Instruments and assessment time points

Depressive symptoms were assessed using the Beck Depression Inventory-II (BDI-II), and anxiety symptoms using the Beck Anxiety Inventory (BAI). PHQ-9 and MADRS data were available for part of the sample, but BDI-II was selected as the primary outcome because it was more consistently available across the participating protocols (35, 36).

The scales were administered as part of routine clinical care, predominantly as self-report measures, with some assessments completed during clinical evaluation by the attending psychiatrist. For the SC protocol, BDI-II and BAI assessments were obtained at baseline, at the sixth treatment session, and at the twelfth session. For the IV protocol, assessments were obtained at baseline and at the sixth session. Assessments were completed up to 24 hours after completion of the sixth session. The 6-session analysis therefore represented the common treatment milestone available for both protocols. Protocol endpoint was defined as the assessment associated with session 6 for IV and session 12 for SC. Because these endpoints occurred after different numbers of administrations and different calendar treatment durations, they should be interpreted as protocol-specific endpoints rather than equivalent treatment-time assessments.

### Outcomes

The primary outcome was categorical antidepressant response based on the percentage change in BDI-II score from baseline to the prespecified protocol endpoint: session 6 for the IV protocol and sessions 6 and 12 for the SC protocol. Response was categorized as nonresponse (<25% reduction), partial response (25% to <50% reduction), or response (≥50% reduction). Secondary outcomes included: for the entire sample, continuous changes in BDI-II and BAI scores from baseline to session 6 and to the protocol-specific endpoint; exploratory factor analysis of baseline symptoms; and documented treatment-emergent adverse events, including dissociation, nausea, transient blood-pressure elevation, and treatment discontinuation when ascertainment was available.

### Safety and tolerability

Safety and tolerability data were retrospectively extracted from clinical charts and routine-care monitoring forms for the 72 participants included in the analytical cohort. Safety ascertainment covered weekly medical consultations and included documented acute clinical events and treatment-related intercurrences occurring during the treatment course. Events of interest included dissociative symptoms, nausea, and transient blood pressure elevations (see Table for details). We identified adverse events through reports and clinical exams. The severity of esketamine-associated adverse events was independently assessed by two investigators according to the Hartwig and Siegel Severity Assessment Scale, based on the available medical-record documentation (37).

### Statistical analysis

Continuous variables are reported as mean and standard deviation or median and interquartile range; categorical variables as n (%). Groups were compared using Student’s t-test or Mann–Whitney U test for continuous variables and Pearson’s chi-square or Fisher’s exact test for categorical variables. Categorical responses were compared between groups through Brant’s test, with proportional odds ratio (OR) and 95% confidence intervals. Significance was set at p < 0.05. Monte Carlo simulation was used when expected cell counts were small. Data were organized in Google Sheets and analyzed in RStudio 2026.01.0 Build 392 with R 4.5.2.

#### Categorical clinical response analysis

Categorical clinical response tiers were evaluated using an unadjusted cumulative-link proportional-odds ordinal logistic regression model fitted via the polr function from the MASS package in R. Response categories were ordered hierarchically according to increasing clinical improvement as failure < partial response < full response. The SC esketamine protocol served as the reference group (0), with the IV protocol evaluated as the active predictor (1); thus, a proportional OR greater than 1 represents higher odds of achieving a superior clinical response tier (i.e., transitioning from failure to partial response, or from partial response to full response) for the IV protocol relative to SC. The proportional-odds (parallel slopes) assumption was formally assessed using the Brant test, which demonstrated no statistically significant violation of the parallel regression assumption (chi-square = 0.010, df = 1, p = 0.920 > 0.050; chi-square = 0.700, df = 1, p = 0.400 > 0.050), supporting the use of a single cumulative OR across response thresholds.

#### Categorical clinical outcome analysis

To evaluate categorical clinical outcomes, we established a two-tiered inferential framework: Primary Categorical Analysis: A cumulative link proportional-odds ordinal logistic regression model (polr) was utilized as the main inferential test for response tiers (failure < partial response < full response). This model leverages the directional clinical hierarchy to estimate the relative odds of achieving a superior outcome tier (OR and 95% CI). Unadjusted Baseline Check: An unadjusted Pearson chi-square test was conducted as a supportive, non-directional omnibus check of categorical independence across cell frequencies. Discrepancies in statistical significance between the omnibus chi-square test and the ordinal model reflect the increased statistical power gained by modeling the ordered hierarchy rather than treating categories as unordered nominal data.

### Continuous depressive and anxiety analysis

Continuous depressive and anxiety symptoms between IV and SC were assessed with the BDI-II and BAI, respectively. These continuous trajectories were analyzed separately from the categorical response outcome to describe changes in symptom burden over time. Continuous change scores were summarized as medians with interquartile ranges (IQRs). The between-group difference in location was estimated using the Hodges–Lehmann estimator with corresponding 95% confidence intervals.

### Exploratory factor analysis

Exploratory factor analysis was conducted on baseline BDI-II and BAI scores using principal axis extraction and oblique (oblimin) rotation to assess latent symptom dimensions.

BDI-II analysis identified cognitive-affective and somatic-neurovegetative factors; BAI analysis identified tension/tremor and autonomic/physiological factors. Interpretation followed psychometric literature showing that BDI-II two-factor structures are common but context-dependent (35). Findings were treated as hypothesis-generating (Table).

AI tools were used only for text editing. Data collection and analysis, and manuscript writing, including graphical elaboration, were performed by the authors themselves.

### Ethical considerations

This study was conducted in accordance with the Declaration of Helsinki and was reviewed and approved (CAAE 00085326.3.0000.8707; Ethics Opinion No. 8.634.900; approved on August 7, 2026). Given the retrospective and exclusively de-identified nature of the medical-record review, with no prospective intervention or patient contact, the ethics committee granted a waiver of study-specific informed consent. The study has maintained confidentiality, data protection, and exclusive scientific use of the data throughout. Separately from this research-consent waiver, written clinical consent for the off-label use of injectable esketamine (IV or SC) was obtained from each patient at the point of care, in accordance with Brazilian Federal Council of Medicine Opinion No. 20/2024 (34). We also considered bioethical considerations specific to off-label parenteral use in psychiatry, including transparency, proportionality, and institutional safeguards (32, 32).

## Results

### General characteristics of the sample

The final sample included 72 patients: 26 treated with IV esketamine and 46 with SC esketamine. **Figure 1** shows screening, eligibility, and inclusion. **Table 1** summarizes baseline characteristics and the complete diagnostic distribution. Groups did not differ significantly in age, sex, education, or marital status. The mean age was 36.9 years, and most participants were female. The prevalence of bipolar depressive episodes was lower in the IV group than in the SC group, whereas recurrent unipolar depression was more frequent in the IV group. However, the distribution of depressive episode type did not differ significantly between groups (**Table 1**).

Oral medication use did not differ significantly between the IV and SC groups for antidepressants (88.5% vs. 97.8%; chi-square (1) = 2.78, p = 0.096), antipsychotics (50.0% vs. 47.8%; chi-square (1) = 0.03, p = 0.859), mood stabilizers (57.7% vs. 65.2%; chi-square (1) = 0.40, p = 0.526), or benzodiazepines (11.5% vs. 15.2%; chi-square (1) = 0.19, p = 0.665). Fisher’s exact tests yielded consistent non-significant results (**Table 2**). The observed mean initial esketamine dose was lower in the SC group than in the IV group (26.9 mg [SD 4.9] vs. 36.2 mg [SD 6.9]; U = 1039.5, p < 0.01). At the final protocol sessions, mean doses were comparable: 60.1 mg (SD 15.0) in the SC group and 60.5 mg (SD 19.5) in the IV group (U = 600, p = 0.990).

**Table 2:** Comparison of medication-class use between IV (n=26) and SC (n=46) groups.

Medication class	IV users, n (%)	IV non-users, n	SC users, n (%)	SC non-users, n	χ²	P	Fisher’s p
Antidepressants	23 (88.5%)	3	45 (97.8%)	1	2.776	0.096	0.131
Antipsychotics	13 (50.0%)	13	22 (47.8%)	24	0.031	0.859	1.000
Mood stabilizers	15 (57.7%)	11	30 (65.2%)	16	0.401	0.526	0.615
Benzodiazepines	3 (11.5%)	23	7 (15.2%)	39	0.188	0.665	0.739

IV, intravenous; SC, subcutaneous. Each patient was counted once within each medication class, regardless of the number of medications used.

### Comorbidities and clinical history

**Table 1** summarizes comorbidities, clinical history, and adverse events. Nearly half the sample had no recorded psychiatric comorbidity. Generalized anxiety disorder was more frequent in the IV group than in the SC group. Other comorbidities did not differ significantly.

A previous suicide attempt was common (62.5%) and did not differ between groups. Psychiatric hospitalization history was also similar. These findings indicate substantial baseline severity, although acute suicidal ideation was not a primary outcome.

### Safety and tolerability

No serious treatment-related events or adverse-event-related discontinuations were documented in the analyzed cohort (**Table 1**). Adverse events included dissociation, which was significantly more frequent in the IV group than in the SC group. In contrast, nausea occurred more frequently in the SC group, although this difference was not statistically significant. Transient blood pressure elevation was also more common in the SC group, with no statistically significant difference between the treatment groups.

### Primary outcomes

#### Clinical categorical response after 6 sessions

After 6 sessions, response distribution favored IV treatment (**Table 3**). In the IV group, 19.23% (n = 5) were classified as failures, 23.08% (n = 6) as partial responses, and 57.69% (n = 15) as responses. In the SC group, the corresponding values were 41.30% (n = 19), 28.26% (n = 13), and 30.44% (n = 14).

**Table 3:** Categorical distribution of clinical response in depressive symptoms (BDI-II items) after 6 sessions, by treatment protocol.

Group	Failure	Partial response	Response	Total
IV (n=26)	5 (19.23%)	6 (23.08%)	15 (57.69%)	26 (36.11%)
SC (n=46)	19 (41.30%)	13 (28.26%)	14 (30.44%)	46 (63.89%)
Total	24 (33.33%)	19 (26.39%)	29 (40.28%)	72 (100%)

Pearson’s chi-square = 5.661; df = 2; p = 0.059. Brant Test: X² = 0.01; df = 1, p = 0.92; Proportional OR (IV/SC): 3.06 (1.22 - 8.00).

Although the overall distribution did not differ significantly between protocols (Pearson’s chi-square (2) = 5.661, p = 0.059), the adjusted proportional-odds ordinal logistic regression model indicated greater odds of an earlier response with IV treatment (OR = 3.06; Brant Test: X² = 0.01, df = 1, p = 0.92; 95% CI: 1.22–8.00) (**Table 3**).

#### Clinical categorical response at protocol endpoint

Categorical response did not differ significantly between protocols, suggesting different temporal trajectories but similar final outcomes (**Table 4**). In the IV group, 19.23% (n = 5) were classified as failures, 23.08% (n = 6) as partial responses, and 57.69% (n = 15) as responses. In the SC group, values were 19.57% (n = 9), 34.78% (n = 16), and 45.65% (n = 21)(**Table 4**).

**Table 4:** Categorical distribution of clinical response in depressive symptoms (BDI-II items) at the respective protocol endpoints, by treatment protocol.

Group	Failure	Partial response	Response	N (%sample)
IV (n=26)	5 (19.23%)	6 (23.08%)	15 (57.69%)	26 (36.11%)
SC (n=46)	9 (19.57%)	16 (34.78%)	21 (45.65%)	46 (63.89%)
Total	14 (19.44%)	22 (30.56%)	36 (50.00%)	72 (100%)

Pearson’s chi-square = 1.227; df = 2; p = 0.541. Brant Test: X² = 0.70; df = 1; p = 0.40; Proportional OR (IV/SC): 1.44 (0.57 - 3.73).

### Secondary outcomes

#### Depressive symptoms

BDI-II scores decreased in both treatment groups (**Figure 2**). **Supplementary Table 2** presents detailed BDI-II analyses. From baseline to session 6, the IV group showed a larger median reduction than the SC group, with median changes of −20.5 (IQR, −25 to −12) and −10.5 (IQR, −18 to −5), respectively. The Hodges–Lehmann median difference was −7.00 points (95% CI, −13.00 to −1.00; p = 0.027; Rosenthal r² = 0.068), indicating significantly greater early improvement under the IV protocol. In the SC group, the additional median change from session 6 to session 12 was −2.5 points (IQR, −8 to 1), which did not reach statistical significance (p = 0.059; Rosenthal r² = 0.078). From baseline to each protocol-specific endpoint, median BDI-II changes were −20.5 (IQR, −25 to −12) in the IV group and −16.0 (IQR, −23 to −7) in the SC group. The corresponding between-group median difference was −3.00 points (95% CI, −9.00 to 3.00; p = 0.405; Rosenthal r² = 0.010), indicating no statistically significant difference in overall depressive-symptom reduction at the non-equivalent protocol-specific endpoints.

**Figure 2:**
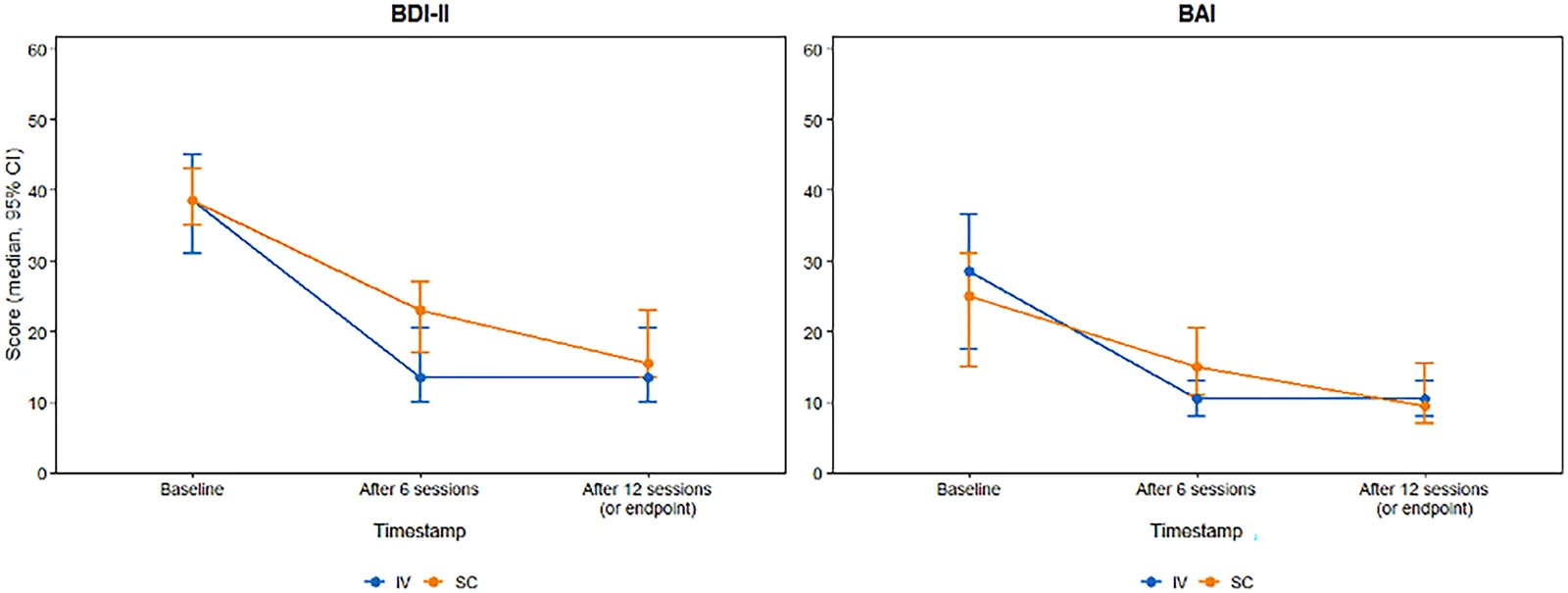
Symptom evolution along IV (n = 26) and SC (n = 46) protocols, assessed by BDI-II and BAI.

#### Anxiety symptoms

BAI trajectories were less consistent than depressive outcomes and were therefore interpreted as complementary and exploratory. **Supplementary Table 3** presents detailed BAI analyses, including factor dimensions. Overall, anxiety improvement was less pronounced than depressive symptom improvement, consistent with the primary treatment indication. Baseline BAI scores were comparable between treatment protocols, with an overall sample median of 25.0 (IQR: 12.0–37.0) (IV median: 28.5, IQR: 14.0–37.0; SC median: 25.0, IQR: 11.0–36.0; p = 0.521). At the common 6-session milestone, the IV group showed a significantly greater reduction in anxiety scores than the SC group (median change: -11.0, IQR: -24.0 to -4.0 vs. -6.0, IQR: -14.0 to 1.0; Hodges–Lehmann median difference was -6.00 (95% CI: -14.00 to 0.00; p = 0.039; Rosenthal r² = 0.059). Within the SC group, anxiety reduction plateaued between sessions 6 and 12, with no additional significant intragroup change (median change: -1.0, IQR: -6.0 to 2.0; p = 0.342; r² = 0.013). At the respective protocol endpoints (session 6 for IV and session 12 for SC), the between-group difference in BAI improvement was no longer statistically significant (median change: -11.0, IQR: -24.0 to -4.0 vs. -7.5, IQR: -16.0 to -3.0). The corresponding between-group median difference was -4.00 (95% CI: -11.00 to 2.00; p = 0.267; Rosenthal r² = 0.017).

#### Exploratory analysis of baseline symptom dimensions

Exploratory factor analysis of baseline BDI-II (**Table 5**) and BAI scores (**Supplementary Materials**) assessed the dimensionality of symptoms. BDI-II analysis yielded two factors explaining 46.0% of variance (27.0% and 19.0%) (**Table 5**). Factor 1 grouped cognitive-affective symptoms, with highest loadings for suicidality (0.80), guilt (0.79), sadness (0.75), pessimism (0.73), low self-esteem (0.72), worthlessness/devaluation (0.71), self-criticism (0.70), past failure (0.63), punishment feelings (0.61), crying (0.47) and agitation (0.40). Factor 2 grouped somatic-neurovegetative symptoms, including appetite changes (0.77), concentration difficulties (0.72), loss of interest (0.70), fatigue (0.63), loss of sexual interest (0.62), low energy (0.59), sleep changes (0.58), indecisiveness (0.53), and loss of pleasure (0.45). Irritability had no sufficient load in any factor. For the BAI, the analysis also revealed a two-factor structure explaining 49.0% of total variance, with factors consistent with tension/tremor symptoms and autonomic/physiological manifestations, as presented in **Supplementary Table 1**.

**Table 5:** Exploratory factor analysis of baseline BDI-II items (n=72 subjects), using principal axis extraction and oblique rotation (oblimin).

Item	Factor 1	Factor 2	Communality
Suicidal Thoughts or Wishes¹	**0.80**	-0.19	0.56
Guilty Feelings¹	**0.79**	0.06	0.66
Sadness¹	**0.75**	-0.04	0.54
Pessimism¹	**0.73**	0.11	0.61
Self-Dislike¹	**0.72**	0.04	0.54
Worthlessness¹	**0.71**	0.00	0.51
Self-Criticalness¹	**0.70**	-0.04	0.48
Past Failure¹	**0.63**	0.05	0.43
Punishment Feelings¹	**0.61**	0.10	0.42
Crying¹	**0.47**	0.31	0.41
Agitation¹	**0.40**	-0.06	0.15
Irritability	0.37	0.13	0.18
Changes in Appetite²	-0.06	**0.77**	0.56
Concentration Difficulty²	-0.24	**0.72**	0.46
Loss of Interest²	0.16	**0.70**	0.59
Tiredness or Fatigue²	-0.05	**0.63**	0.38
Loss of Interest in Sex²	0.06	**0.62**	0.41
Loss of Energy²	0.15	**0.59**	0.44
Changes in Sleeping Pattern²	0.03	**0.58**	0.34
Indecisiveness²	0.29	**0.53**	0.47
Loss of Pleasure²	0.34	**0.45**	0.42
Eigenvalues	5.65	3.91	
% of variance	27.00%	19.00%	

Sample: 72 patients. Factor loadings ≥ 0.40 are highlighted. Total explained variance = 46.0%. ¹Factor 1: cognitive-affective symptoms; ²Factor 2: somatic-neurovegetative symptoms; KMO 0.84; Bartlett’s χ²(210) = 908.61, p < 0.001; Factor-retention method: Parallel Analysis with Oblique Rotation (Oblimin); Factor correlations: r = 0.53; Communalities: h² range = 0.15-0.66 (in table as a column); Stability limitations: Sample-size (n = 72) to Items (BDI-II = 21 items) ratio 3.43:1, low-to-high communalities range 0.15-0.66. No missing-data handling procedure (e.g., imputation or case exclusion) was required for the factor analyses.

Factor loadings ≥ 0.40 are highlighted. Highlighted values are sufficiently correlated with the corresponding factor.

## Discussion

### Main findings

This multicenter retrospective study compared 2 center-specific parenteral esketamine treatment protocols in patients with TRD. At the common 6-session assessment, patients treated under the IV protocol showed a more favorable categorical response distribution, whereas categorical outcomes at the respective protocol endpoints—session 6 for IV and session 12 for SC—were not statistically distinguishable. Additionally, exploratory factor analysis revealed that baseline depressive symptoms clustered into distinct cognitive-affective and somatic-neurovegetative dimensions. Taken together, these findings underscore the value of real-world studies in characterizing esketamine response trajectories and suggest that the primary divergence between IV and SC routes may rely on response speed rather than overall antidepressant efficacy, in line with prior literature (5, 23, 25, 38, 39).

### Protocol characteristics

Both protocols were clinically manageable for the eligible sample, with no severe adverse events or discontinuations due to adverse events. Recorded effects—transient dissociation, nausea, and mild hypertensive peaks—match known safety profiles (1, 24, 25, 40, 41). Dissociation was significantly more frequent with IV treatment, a practical concern for patient experience, monitoring, staffing, and workflow. Although safety equivalence cannot be inferred, both routes appeared tolerable under structured monitoring (24, 25, 40).

Initial dose differences may have influenced early response trajectories, as the SC group started at a lower mean dose than the IV group. Although final mean doses converged toward a similar range, equivalent therapeutic exposure cannot be inferred due to route, cumulative exposure, treatment duration, and center-specific procedural variations. Future prospective studies with standardized dosing and titration protocols are needed to isolate route-specific effects.

Baseline clinical characteristics warrant careful consideration. Although both cohorts had comparable demographic profiles in age, sex distribution, educational level, and marital status, diagnostic composition differed notably. Recurrent major depressive disorder was predominantly represented among patients under the IV protocol, whereas those receiving SC administration displayed a broader diagnostic variation. Furthermore, comorbid generalized anxiety disorder occurred at a higher frequency in the IV cohort. These naturalistic baseline differences may explain observed variations in therapeutic response, side-effect profiles, and clinical decision-making regarding route selection.

### Clinical implications

Overall, the results align well with the existing IV ketamine and esketamine literature. As the most thoroughly investigated parenteral approach, IV administration consistently provides rapid clinical benefit across controlled trials and naturalistic TRD cohorts (1, 7, 40, 41). Because substantial improvement frequently emerges within the initial infusions, the IV route remains particularly suitable when rapid onset is critical (20, 38, 39). The meta-analysis of RCTs found that patients were 7 times more likely to show a clinical response 24 hours after ketamine infusion than after placebo infusion (20). Still, some early non-responders benefit from continued treatment, highlighting the distinction between early and final response (38, 39).

Conversely, SC administration has attracted growing interest because of its feasibility and operational simplicity (24). The emerging subcutaneous literature adds context to this interpretation. George et al. found individually titrated SC esketamine well tolerated and effective in older adults with TRD. The KADS trial suggests that response-guided dose escalation may be relevant to subcutaneous treatment, as flexible dosing between 0.5 and 0.9 mg/kg was associated with greater efficacy than a fixed 0.5 mg/kg regimen in a randomized active-controlled study of racemic ketamine (42). Similarly, the open-label study of repeated subcutaneous esketamine by Palhano-Fontes et al. reported clinically meaningful improvement after 8 weekly sessions, supporting the possibility that continued, individualized treatment may benefit patients who do not respond early (30). We should weigh the time difference against tolerability, especially the higher dissociation frequency with IV treatment. In non-emergency outpatient settings, SC treatment may be attractive when convenience, tolerability, and feasibility are priorities. However, these studies cannot establish that longer treatment or titration explains the trajectory observed in our cohort, but they provide a relevant framework for interpreting the additional improvement observed under the 12-session SC protocol.

### Clinical trials versus real-world studies

This study also addresses the gap between trial efficacy and real-world feasibility. Trials prioritize standardization; clinical practice must adapt to resources, tolerability, patient characteristics, and service organization (18, 23, 29). At the common 6-session assessment, the observed categorical response distribution differed between the two protocols, whereas no statistically significant difference in categorical response was detected at their respective endpoints. Because end points represented different treatment durations and cumulative exposures, these findings do not demonstrate faster route-specific action or equivalent final effectiveness. Likewise, the higher recorded frequency of dissociation in the IV group should be interpreted in the context of differences in dose, administration procedure, monitoring, treatment exposure, and center-specific documentation rather than as an isolated route-specific tolerability effect. Still, current results support the hypothesis of SC esketamine as a plausible alternative in this care context, especially given its easier implementation, gradual trajectory, and protocol-related differences (18, 24, 40, 41).

The high prevalence of previous suicide attempts reinforces baseline severity but does not demonstrate antisuicidal effectiveness because acute suicidal ideation was not a primary outcome. Evidence on ketamine/esketamine and suicidality remains heterogeneous; IV ketamine has stronger support for rapid reductions in suicidal ideation than esketamine across formulations and routes (10). Future IV–SC studies should include standardized suicidality measures.

Finally, anxiety outcomes were less consistent than depressive outcomes, likely because the primary indication was TRD. Ketamine’s strongest replicated effects concern depressive symptoms and, in some contexts, suicidal ideation; anxiolytic effects are more variable (4, 5, 7) The higher generalized anxiety disorder frequency in the IV group further complicates interpretation. Anxiety response should therefore be treated as secondary.

### Exploratory analysis of baseline symptom dimensions

Exploratory factor analysis added a psychopathological dimension. Baseline depressive symptoms clustered into cognitive-affective and somatic-neurovegetative dimensions. Suicidality loaded with guilt, sadness, pessimism, devaluation, and self-criticism, suggesting a broader depressive core rather than an isolated feature (4, 5). This interpretation must remain cautious. The factor analysis was exploratory and does not show that the cognitive-affective cluster predicts esketamine response or defines a specific indication profile. It only indicates coherent baseline symptom organization. Larger prospective studies should test whether baseline domains predict IV or SC response trajectories (24, 35).

BDI-II structure is not fixed across settings. Psychometric reviews show that two-factor cognitive-affective and somatic-vegetative solutions are common, but item distributions and loadings vary across populations, contexts, and languages (35). Our findings should therefore be viewed as clinically useful within this sample, not definitive across settings.

### Bioethical considerations

Our results highlight the need for standardization, transparency, and monitoring of off-label SC and IV esketamine across Brazilian services (33). Parenteral ketamine/esketamine outside approved formulations may be justified in severe refractory cases but require informed consent, documentation, risk-benefit assessment, and institutional safeguards (32, 32, 34). Brazilian guidance emphasizes autonomy, beneficence, non-maleficence, and justice (32, 32). CFM Opinion No. 20/2024 states that parenteral esketamine for mental disorders is deemed off-label, should be reserved for insufficient response to established treatments, requires written consent and chart documentation, and must occur in monitored settings under physician responsibility (34). Continuity of care is also ethical. An acute response without a realistic maintenance plan may create inequity. Community ketamine programs have noted that the inability to sustain treatment after initial response raises justice concerns (18, 41). Consequently, by favoring equitable access while maintaining rigorous monitoring, SC ketamine may offer clinical benefits exceeding symptom improvement (18, 32, 32, 34, 41).

### Limitations and strengths

This study has limitations that deserve further commentary. Its retrospective naturalistic design limits causal inference and increases indication bias. Groups came from different centers, followed different routines, and received non-equivalent protocols, so the comparison reflects real-world institutional strategies rather than standardized interventions. Thus, the absence of an endpoint difference should not be interpreted as equivalence or non-inferiority, because this study was not designed for that purpose. Diagnostic heterogeneity limits subgroup inference. In addition, the relatively small sample size prevented a more refined statistical workup, including thoroughful exploratory factor analysis. Adverse events were derived from routine clinical documentation rather than prospectively solicited using a standardized safety instrument, and formal severity grading or serious-adverse-event adjudication was not uniformly available. Standardized safety follow-up was unavailable for the 54 patients who discontinued; cognitive outcomes, acute suicidality, maintenance outcomes, and economic data were unavailable. Factor analyses were conducted on a modest sample and remain exploratory. Intranasal esketamine was not evaluated but remains relevant. It has the strongest regulatory support and evidence for efficacy and relapse prevention (1, 16, 41). Yet cost, limited availability, and supervised-administration requirements continue to restrict access (18, 40, 41).

The study also has strengths. It aligns with other real-world studies reporting clinical outcomes under distinct treatment protocols (18, 23–25, 29, 35, 40) and is among the few multicenter Latin American real-world comparisons of IV and SC esketamine. These findings inform clinical recommendations regarding supervised administration, objective symptom assessment, response-guided protocols, and rigorous monitoring during routine psychiatric ketamine-based care (43). By integrating clinical outcomes, dosing regimens, tolerability profiles, comorbidities, and baseline symptom structures, this work provides a comprehensive, practice-oriented perspective.

## Concluding remarks

In this multicenter real-world cohort, patients treated under two center-specific parenteral esketamine protocols showed different observed response distributions over treatment. At the common 6-session assessment, the categorical response distribution was more favorable in the IV group, whereas categorical outcomes at the respective, non-equivalent protocol endpoints were not statistically distinguishable, although these endpoints occurred after different numbers of sessions. The SC group achieved similar final categorical outcomes after a longer protocol and exhibited a lower recorded frequency of dissociation. Because the administration route, center, dosing strategy, treatment duration, diagnostic composition, and routine-care procedures were intertwined, these findings cannot determine which factors accounted for the observed differences. Consequently, upcoming studies with parenteral esketamine should employ harmonized eligibility criteria, dosing regimens, and assessment protocols to ascertain potential route-specific differences in real-world scenarios.

## Data Availability

The original contributions presented in the study are included in the article/**Supplementary Material**. Further inquiries can be directed to the corresponding author.

## Supplementary material

Supplementary Table 1 Exploratory factor analysis of baseline BAI items (n=72 subjects), using principal axis extraction and oblique rotation (oblimin). Sample: 72 patients. Factor loadings ≥ 0.40 are highlighted. Total explained variance = 49.0%. ¹Factor 1: tension/tremor; ²Factor 2: autonomic/physiological factors. KMO 0.85; Bartlett’s χ² (210) = 850.75, p < 0.001; Factor-retention method: Parallel Analysis with Oblique Rotation (Oblimin); Factor correlations: r = 0.47; Communalities: h² range = 0.12-0.73 (in table as a column); Stability limitations: Sample-size (n = 72) to Items (BAI = 21 items) ratio 3.43:1, low-to-high communalities range 0.12-0.73. No missing-data handling procedure (e.g., imputation or case exclusion) was required for the factor analyses.

Supplementary Table 2 BDI-II score improvement between groups.

Supplementary Table 3 BAI score improvement between groups.

Supplementary Figure 1 Distribution of BAI scores/dimensions tension/tremor (left) and autonomic/physiological factors (right) at baseline.

Supplementary Figure 2 Distribution of BAI dimensions tension/tremor (left) and autonomic/physiological factors (right) after 6 treatment sessions with esketamine IV and SC.

Supplementary Figure 3 Distribution of the BAI scores/dimensions tension/tremor (left) and autonomic/physiological factors (right) at the endpoint of the respective protocols.
