# Supplementary material for: Real-world clinical outcomes under intravenous and subcutaneous esketamine protocols for treatment-resistant depression: a multicenter, retrospective study
Source: Front Psychiatry. 2026 Oct 16;17:1963841. doi: 10.3389/fpsyt.2026.1963841 (PMC13720280; doi:10.3389/fpsyt.2026.1963841)
Supplement: Supplementary file 1 [file SupplementaryFile1.docx]

### SUPPLEMENTARY MATERIALS

**Supplementary Table 1. Exploratory factor analysis of baseline BAI items (n=72 subjects), using**

### principal axis extraction and oblique rotation (oblimin).

| **Item** | **Factor 1** | **Factor 2** | **Communality** |
| --- | --- | --- | --- |
| Wobbliness in Legs¹ | **0.84** | -0.12 | 0.63 |
| Hands Trembling¹ | **0.83** | -0.14 | 0.60 |
| Scared¹ | **0.80** | 0.11 | 0.73 |
| Shaky¹ | **0.78** | -0.19 | 0.50 |
| Terrified or Afraid¹ | **0.69** | 0.26 | 0.71 |
| Nervous¹ | **0.66** | 0.19 | 0.59 |
| Fear of Worst Happening¹ | **0.59** | 0.13 | 0.44 |
| Fear of Dying¹ | **0.57** | -0.12 | 0.27 |
| Feeling of Choking¹^,^ ² | **0.49** | **0.45** | 0.64 |
| Fear of Losing Control¹ | **0.46** | 0.37 | 0.52 |
| Difficulty Breathing¹ | **0.46** | 0.40 | 0.55 |
| Feeling Insecure¹ | **0.44** | 0.27 | 0.37 |
| Unsteady | 0.39 | 0.35 | 0.40 |
| Face Flushed² | -0.17 | **0.81** | 0.56 |
| Feeling Hot² | -0.06 | **0.72** | 0.48 |
| Sweating (Not Due to Heat)² | 0.17 | **0.67** | 0.59 |
| Indigestion or Discomfort in  Abdomen² | -0.01 | **0.66** | 0.43 |
| Heart Pounding / Racing² | 0.20 | **0.63** | 0.55 |
| Numbness or Tingling² | 0.10 | **0.48** | 0.28 |
| Unable to Relax² | 0.24 | **0.42** | 0.33 |
| Faint / Lightheaded | 0.12 | 0.27 | 0.12 |
| Eigenvalues | 5.94 | 4.36 |  |

| % of variance | 28.00% | 21.00% |  |
| --- | --- | --- | --- |

Note. Sample: 72 patients. Factor loadings ≥ 0.40 are highlighted. Total explained variance = 49.0%. ¹Factor 1: tension/tremor;

²Factor 2: autonomic/physiological factors. *KMO 0.85; Bartlett’s χ² (210) = 850.75, p < 0.001; Factor-retention method:*

*Parallel Analysis with Oblique Rotation (Oblimin); Factor correlations: r = 0.47; Communalities: h² range = 0.12-0.73 (in*

*table as a column); Stability limitations: Sample-size (n = 72) to Items (BAI = 21 items) ratio 3.43:1, low-to-high communalities*

*range 0.12-0.73. No missing-data handling procedure (e.g., imputation or case exclusion) was required for the factor analyses.*

### Supplementary Table 2. BDI-II score improvement between groups

**Median (IQR) BDI-II Improvement**

**Time Interval**

IV (n=26)

SC (n=46)

**Median Difference (95%CI)¹	P	r²**

Baseline to Session 6	-20.5 (-25 - -12)	-10.5 (-18 - -5)	-7.00 (-13.00 - -1.00)	**0.027**	0.068

Session 6 to Session 12*	-	-2.5 (-8 - 1)	2.37 (0.00 - 6.00)	0.059	0.078

Baseline to Endpoint	-20.5 (-25 - -12)	-16.0 (-23 - -7)	-3.00 (-9.00 - 3.00)	0.405	0.010

*: Applies only for SC group (intra-group analysis). Session 6 is also the Endpoint for EV group. ¹Hodges-Lehmann; ²Rosenthal.

### Supplementary Table 3. BAI score improvement between groups

**Median (IQR) BAI Improvement**

**Time Interval**

IV (n=26)

SC (n=46)

**Median Difference (95%CI)¹	P	r²**

Baseline to Session 6	-11 (-24 - -4)	-6 (-14 - 1)	-6.00 (-14.00 - 0.00)	**0.039**	0.059

Session 6 to Session 12*	-	-1 (-6 - 2)	1.00 (-1.00 - 5.00)	0.342	0.013

Baseline to Endpoint	-11 (-24 - -4)	-7.5 (-16 - -3)	-4.00 (-11.00 - 2.00)	0.267	0.017

*: Applies only for SC group. Session 6 is also the Endpoint for IV groups. ¹Hodges-Lehmann; ²Rosenthal.

**Supplementary Figure 1. Distribution of BAI scores/dimensions tension/tremor (left) and**

**autonomic/physiological factors (right) at baseline.**

**Supplementary	Figure	2.	Distribution	of	BAI	dimensions	tension/tremor	(left)	and**

**autonomic/physiological factors (right) after 6 treatment sessions with esketamine IV and SC.**

### Supplementary Figure 3. Distribution of the BAI scores/dimensions tension/tremor (left) and

**autonomic/physiological factors (right) at the endpoint of the respective protocols**.
